# Supplementary material for: Integrated analysis of DNA methylation profile of HLA-G gene and imaging in coronary heart disease: Pilot study
Source: PLoS One. 2020 Aug 13;15(8):e0236951. doi: 10.1371/journal.pone.0236951 (PMC7425923; doi:10.1371/journal.pone.0236951)
Supplement: S1 Table — (DOCX) [file pone.0236951.s001.docx]

**Supplementary Table 1. Correlation analysis between CCTA variables and methylation levels on quantitative imaging features**

|  | **HLAG_B_meth** | **HLAG_C_meth** | **HLAG_F_meth** |
| --- | --- | --- | --- |
| **Ca score*** | Rho=0.16, p= 0.57 | Rho=-0.17, p= 0.80 | **Rho=0.57, p= 0.03** |
| **Plaque composition** | R=-0.11, p=0.700 | R=-0.043, p=0.89 | R=-0.32, p=0.27 |
| **N° of Plaque segments** | R=0.025, p=0.93 | R=0.0056, p=0.99 | R=0.33, p=0.25 |
| **N° of Stenotic vessels** | R=0.17, p=0.57 | R=-0.0029, p=0.99 | R=0.39, p=0.17 |

*Continuous variable; Rho: Spearman’s rank coefficient; p: p-value; R: regression coefficient.
