# Supplementary figures and images for: Integrated analysis of DNA methylation profile of HLA-G gene and imaging in coronary heart disease: Pilot study
Source: PLoS One. 2020 Aug 13;15(8):e0236951. doi: 10.1371/journal.pone.0236951 (PMC7425923; doi:10.1371/journal.pone.0236951)

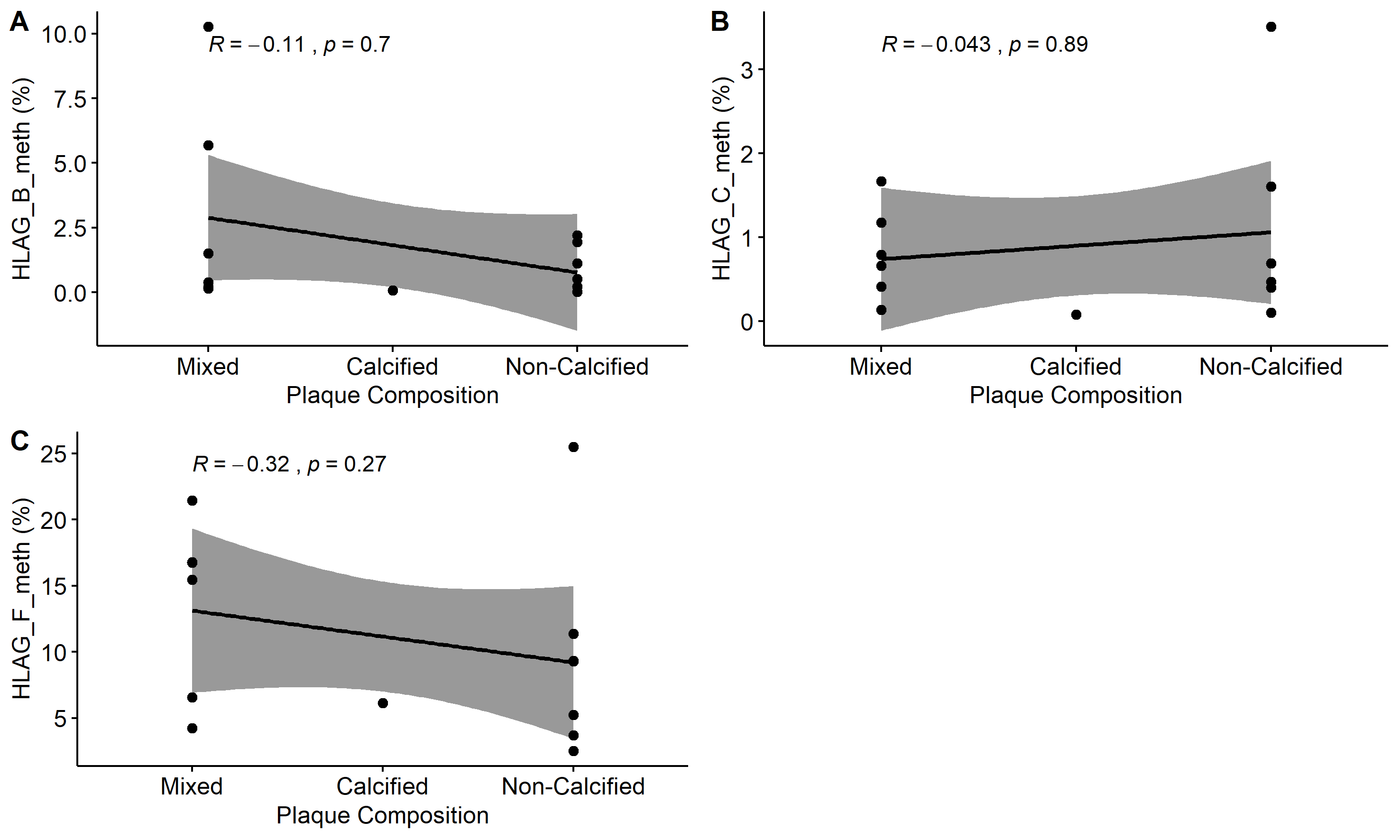

Supplement: S1 Fig — Regression Line for Plaque composition. (A-C) HLA-G nucleotides methylation levels (B, C, F) against the plaque composition variables. (TIFF) [file pone.0236951.s002.tiff]

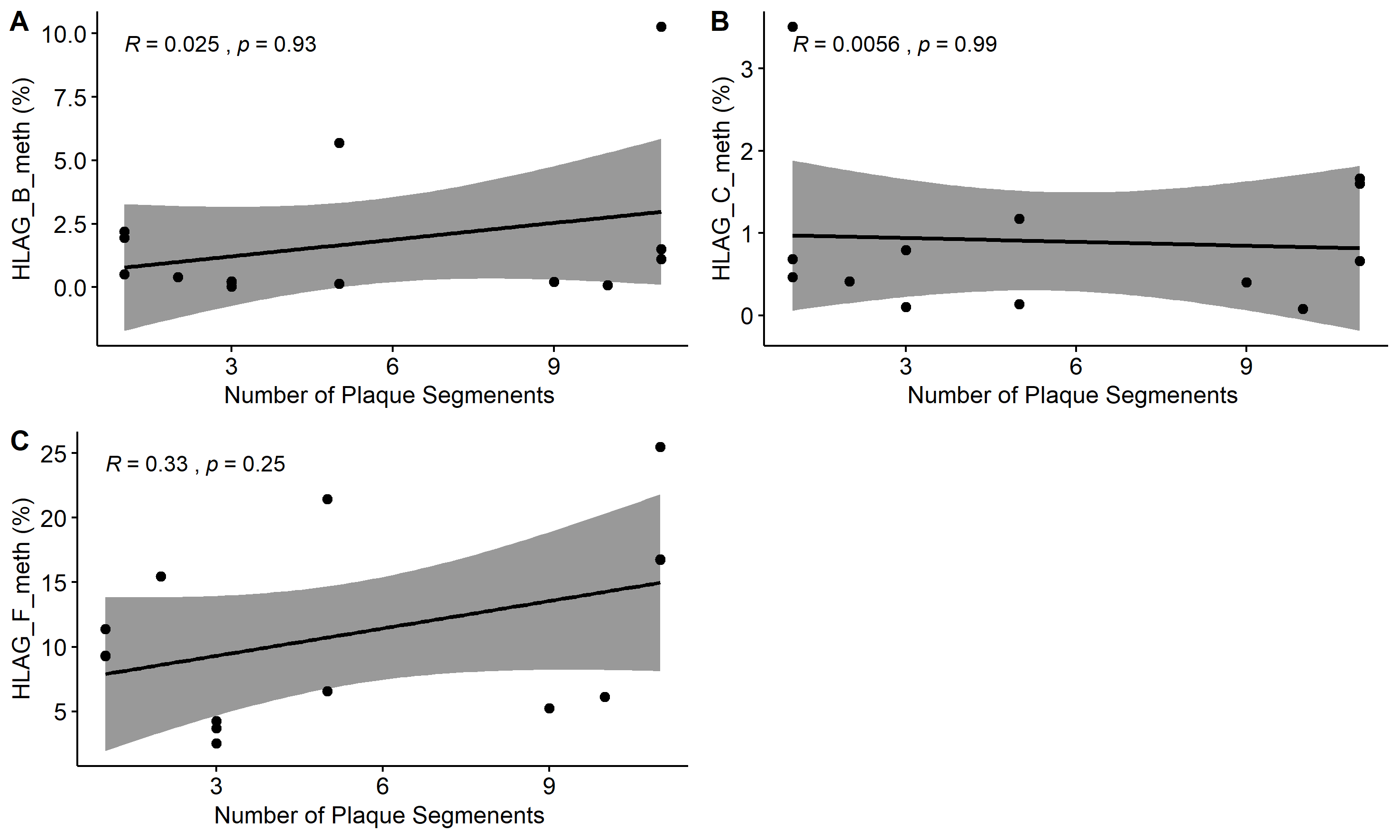

Supplement: S2 Fig — Regression Line for Number of Plaque segments. (A-C) HLA-G nucleotides methylation levels (B, C, F) against the number of plaque segments. (TIFF) [file pone.0236951.s003.tiff]

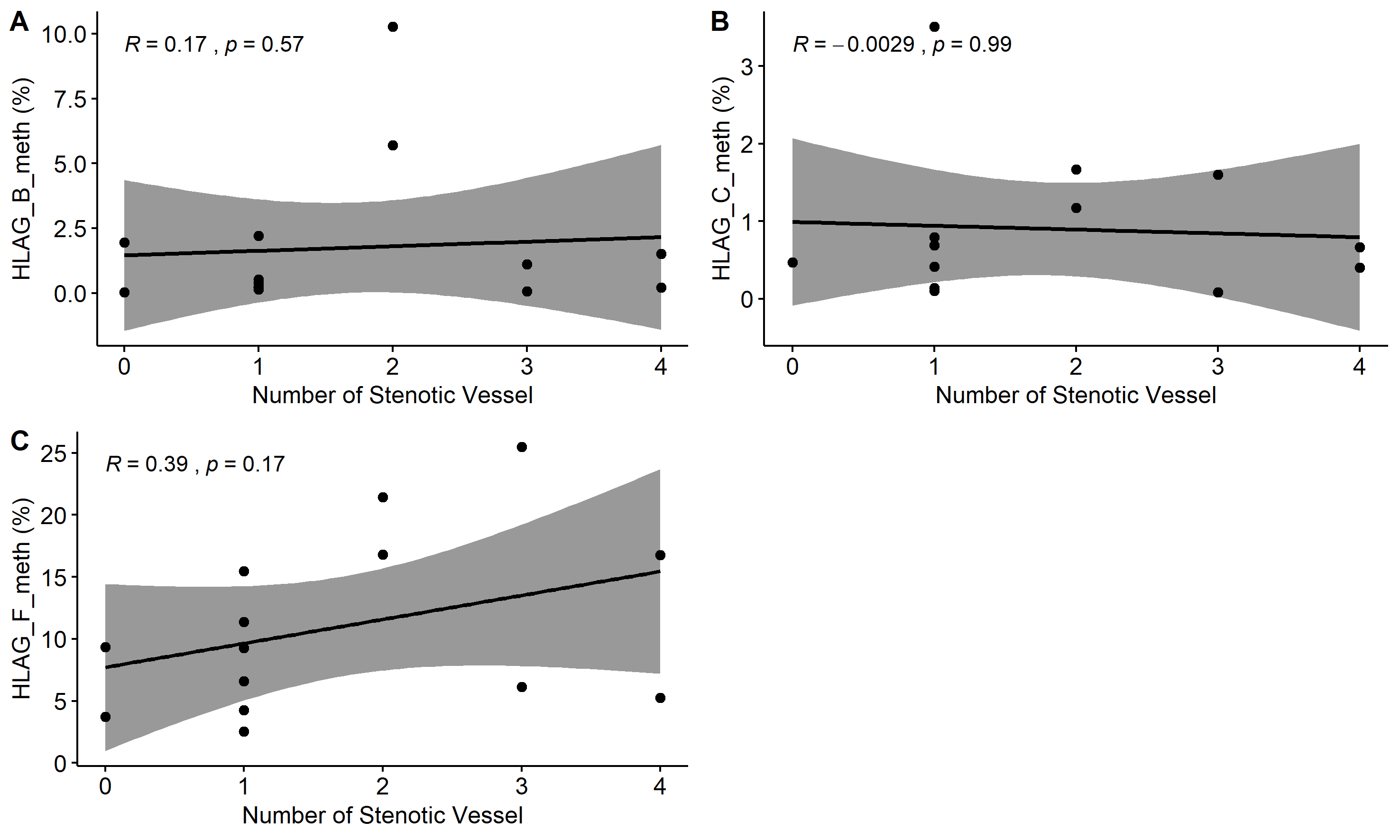

Supplement: S3 Fig — Regression Line for Number of stenotic vessels. (A-C) HLA-G nucleotides methylation levels (B, C, F) against the number of stenotic vessels. (TIFF) [file pone.0236951.s004.tiff]
