# Supplementary material for: Integrated analysis of DNA methylation profile of HLA-G gene and imaging in coronary heart disease: Pilot study
Source: PLoS One. 2020 Aug 13;15(8):e0236951. doi: 10.1371/journal.pone.0236951 (PMC7425923; doi:10.1371/journal.pone.0236951)
Supplement: Attachment — Submitted filename: Response to Reviewers.docx [file pone.0236951.s005.docx]

**PONE-D-20-07217**

**Integrated analysis of DNA methylation profile of HLA-G gene and imaging in coronary heart disease: pilot study**

|  | **Comments** | **Answers** |
| --- | --- | --- |
| Reviewer #1: | Interesting paper and results, with the limitations of a pilot study.  It will be interesting to see if this test can be used to predict severity of disease and evaluate use evaluating prognostic value. | We thank the reviewer for this comment.  We agreed with the reviewer. Therefore, we investigated the prognostic value of the statistically significant methylation levels to predict the severity of disease (critical stenosis) via Generalized linear model (GLM).  The performance was evaluated using a 3-fold cross validation. The best performance was achieved using 5’UTR-CpG of HLAG_F methylation level. Data were reported in a new Table (Table 5). |
| Reviewer #2: | This study investigated DNA methylation level of the unique CpG island of HLA-G gene in CHD patients and evaluated the correlation with cardiac computed tomography angiography (CCTA) features. It is interesting and of clinical significance. | We thank the reviewer for these comments. |
|  | 1. In the section of in Introduction, the author should express why choose the DNA methylation as the study Aims and write the passage more logistic. | In according with this suggestion, in order to clarify the choice of DNA methylation as first analysis approach, we re-paraphrased Introduction section. |
|  | 2. Table 1 in the “2.2 Patient selection” can be deleted， for it is same as table1 in the result. | As suggested, we preferred to insert the Table titled “Baseline Characteristics of patients with non-critical stenosis (NCS) and critical stenosis (CS)” only in the Methods and rename it in the Results section. |
|  | 3. For gender, non-critical stenosis (NCS) and critical stenosis (CS) was differently, the authors need to take subgroup analysis in the section of comparing DNA methylation level and evaluated the correlation with cardiac computed tomography angiography (CCTA) features | We agree with the reviewer. However, due to the limitation of the pilot study, a subgroup analysis was not possible at this moment. As reported in Table 1, in the critical stenosis group only one female is included. Therefore, it was not possible to evaluate the correlation in this group. |
|  | 1. Table 3, the difference methylation level between non-critical stenosis (NCS) and critical stenosis (CS) was close to each other， so the result should be taken cautiously. | We agree with the review and this aspect was better highlighted in the limitation of the study. |
|  | 2. Table 3, the authors need to add Control group | In according with the reviewer, we decided to include Control group (NCS) also if several values for imaging parameters were about zero. We added this column in the Table 3 for the final version of the text, as suggested. |
|  | 3. The author should add the analysis of mRNA expression, the investigate the relationship between mRNA expression and methylation level | We thank the reviewer for this suggestion. Unfortunately, we have disposable only plasma, but no RNA samples for these patients. In order to validate our methylation gene results, we decided to analyze HLA-G plasma concentration levels, as already previously reported in the manuscript. In addition, here, we calculated the relationship between methylation grade and plasma concentrations. Finally, statistically significant methylation levels were investigated in a graphical inspection via Boxplot.  The new Figure (Figure 5) was reported in the final version of the manuscript. |
